# Investigation of sphingolipid-related genes in lung adenocarcinoma

**DOI:** 10.3389/fmolb.2025.1548655

**Published:** 2025-03-13

**Authors:** Jibin Mao, Li Li, Hui Sun, Jie Han, Jinqiao Li, Chang-Sheng Dong, Hongyu Zhao

**Affiliations:** ^1^ Department of Radiation Oncology, Affiliated Hospital of Nantong University, Medical School of Nantong University, Nantong, China; ^2^ Department of Radiation Oncology, The Affiliated Hospital of Nantong University, Nantong, China; ^3^ Department of Pathology, The Affiliated Hospital of Nantong University, Nantong, China; ^4^ Cancer Institute of Traditional Chinese Medicine/Department of Oncology, Longhua Hospital, Shanghai University of Traditional Chinese Medicine, Shanghai, China

**Keywords:** sphingolipid metabolism, lung adenocarcinoma, prognostic signature, immune microenvironment, immunohistochemical experiment *in vitro*

## Abstract

**Background:**

Lung adenocarcinoma (LUAD) is responsible for majority cases of lung cancer and considered to be the primary cause of cancer-related mortality. The imbalance of cellular proliferation and apoptosis is critically implicated in the pathogenesis and progression of LUAD. Sphingomyelin, a vital lipid component, is integral to the regulation of tumor cell growth and apoptosis, and has garnered significant attention as a target in novel anticancer therapies. The pivotal molecules involved in sphingomyelin metabolism are crucial in modulating tumor cell behavior, thereby influencing clinical outcomes.

**Methods:**

A comprehensive consensus clustering analysis was conducted by collecting clinical LUAD figures from the TCGA and GEO databases. By employing Cox regression and Lasso regression analysis, a prognostic model for LUAD patients was established by identifying seven sphingolipid-related genes (SRGs), and validated in the GEO database. The study also delved into the clinical relevance, functional capabilities, and immune implications of prognostic signals associated with sphingolipid metabolism. Finally, experiments conducted *in vitro* confirmed the imbalance of sphingolipid-associated genes in LUAD.

**Results:**

Using the prognostic model, lung adenocarcinoma (LUAD) patients can be divided into high-risk and low-risk groups. Meanwhile, we can observe marked disparities in survival times among these groups. Additionally, the model demonstrates high predictive accuracy in external validation cohorts. Research on the immune microenvironment and immunotherapy points to this risk stratification as a useful reference for immunotherapeutic strategies in LUAD. Finally, our hypothesis was corroborated through *in vitro* experiments.

**Conclusion:**

This study demonstrates that sphingolipid-related gene prognostic characteristics correlate with tumor progression and recurrence, long-term prognosis, and immune infiltration in LUAD patients. The outcomes of our study could help shape innovative strategies for early intervention and prognosis prediction in lung adenocarcinoma.

## 1 Introduction

Lung cancer ranks first among cancers all year round, while the 5-year survival rate is only 26% (Bray et al., 2024). Lung adenocarcinoma (LUAD), as the most common form of NSCLC, comprises approximately 40% of lung cancer cases (Leiter et al., 2023). Traditional lung cancer treatments primarily include surgery, chemotherapy, and radiotherapy (Miller et al., 2022). Although these techniques have made rapid progress in these years, patients with lung adenocarcinoma have an unsatisfactory prognosis. Recent advancements in immunotherapy and targeted therapy have introduced a novel approach to precise treatment for lung cancer patients. For example, some targeted drugs like EGFR tyrosine kinase inhibitors, including gefitinib and erlotinib, are extensively utilized in patients with certain gene mutations (Roviello, 2015; Lo Sardo et al., 2018). The use of immune checkpoint inhibitors (ICIs) leads to a marked improvement in survival rates for those with locally advanced or metastatic NSCLC (Forde et al., 2022). Nevertheless, few patients have experienced the anticipated advantages of ICI therapy (Sharma et al., 2017). Identifying potential biomarkers to predict lung adenocarcinoma’s response to chemotherapy, targeted therapy, and immunotherapy holds significant clinical importance. Traditional clinical models forecast LUAD prognosis using tumor extension, performance status, pathological staging, and TNM staging indicators. However, these models have struggled to produce satisfactory outcomes due to the heterogeneity in LUAD (Zuo et al., 2019). Consequently, novel models must be developed for LUAD treatment and prognosis.

Metabonomics has recently been acknowledged for its substantial influence on lung cancer development and progression (Kerk et al., 2021; Brock Malcolm et al., 2008; Wang et al., 2019). Sphingolipids, a crucial class of lipids, have emerged as a focal point of research. Sphingolipid metabolism is closely associated with cellular processes like proliferation, apoptosis, necrosis, and autophagy, supporting the study of various physiological and pathological conditions (Ogretmen, 2018; Breslow and Weissman, 2010; Kraft, 2016). Sphingolipid metabonomics constitutes a vital component of cellular signal transduction plays a pivotal role in Immune cell recruitment and function regulation in tumor microenvironment (Ogretmen, 2018). The key components of sphingolipids are Ceramide, SM (Sphingomyelin), sphingosine-1-phosphate (S1P),glycosphingolipid (GSL) and various sphingolipids, including simple or complex gangliosides (Mu et al., 2024). Ceramide (Cer) is known to regulate cell aging, apoptosis and induce cell cycle arrest (Obeid et al., 1993), whereas S1P is associated with promoting proliferation and exhibiting anti-apoptotic properties (Lee et al., 1998). Sphingolipids with biological activity have become essential regulators in cancer cell biology. S1P is associated with cell survival, angiogenesis, chemotherapy resistance, and cancer cell invasion. Conversely, ceramides are linked to apoptosis induction, growth inhibition, enhanced sensitivity to chemotherapy, and the promotion of senescence in cancer cells (Hannun et al., 2015; Morad and Cabot, 2013). Furthermore, ceramides and S1P are crucial signaling molecules involved in fundamental cellular processes, including Cell inflammation, proliferation, vascular endothelial barrier, cell transport, stress, autophagy, death and so on (Dany and Ogretmen, 2015; Pettus et al., 2005).

Sphingolipid metabolites have been shown in recent research to be key components of immunotherapy for NSCLC (Wang et al., 2023; Zhang et al., 2023) and can influence the initiation and progression of lung adenocarcinoma via autophagy and apoptosis mechanisms (Shweta et al., 2022). Research by Gokhan Unlu et al. suggests that sphingolipid synthesis may allow tumor cells to escape immune detection by NK and CD8^+^ T cells and resist IFNγ signaling effects (Soula et al., 2024). Nonetheless, the association between sphingolipid metabolism and the biological and clinical outcomes of lung adenocarcinoma remains insufficiently elucidated. Therefore, identifying the precise mechanisms and targeted therapies is necessary for the diagnosis and treatment of lung adenocarcinoma. Further research is necessary to understand how sphingolipid-related genes (SRGs) can predict the therapeutic and long-term prognosis for patients.

In this research, we obtained publicly available lung adenocarcinoma datasets from the TCGA and GEO databases. We created a novel prognostic model incorporating seven sphingolipid-related genes utilizing comprehensive bioinformatics analyses. Based on risk stratification, patients with lung adenocarcinoma were categorized into high and low-risk groups. Furthermore, the alterations in immune infiltration and immune checkpoint expression in these patients were assessed through the sphingolipid metabolism spectrum. Ultimately, we performed *in vitro* experiments and confirmed the prognostic significance of the key genes in our model using the Nantong Cohort. Our study introduces an innovative method for diagnosing and treating lung adenocarcinoma (LUAD).

## 2 Materials and methods

### 2.1 Data collection of LUAD patients

All mRNA transcriptome data, survival information, and clinical characteristics were obtained from The Cancer Genome Atlas (TCGA) database through the UCSC Xena platform (http://www.genome.ucsc.edu/). Following the process of data screening and removal of irrelevant information, a total of 501 cancerous tissues and 59 adjacent non-cancerous tissues were included in the study. A combined dataset of 331 patients, including mRNA expression data and survival time, was sourced from the Gene Expression Omnibus (GEO) database, specifically from GSE31210 (n = 246) and GSE30219 (n = 85).

### 2.2 Acquisition of genes related to sphingolipids

GeneCards were selected as the source of genes related to sphingolipid metabolism, 721 SRGS with correlation scores greater than the median (3.8464) were selected for follow-up studies.

### 2.3 Consensus clustering

The “Consensus ClusterPlus” R package was utilized for unsupervised clustering. A consensus clustering method using the K-means algorithm with Euclidean distance was executed 1,000 times, employing an 80% resampling rate. The ideal number of clusters was ascertained by employing an empirical cumulative distribution function diagram.

### 2.4 Establishment of a risk signature associated with sphingolipid

Sphingolipid-related genes with prognostic significance were initially identified by us through a univariate Cox analysis. The LASSO algorithm was subsequently utilized in the SRGs in the TCGA-LUAD cohort. Finally, through the stepwise Cox regression algorithm, a sphingolipid-related signature was established. Consequently, the algorithm can assign a risk score to each LUAD. Patients within the TCGA-LUAD cohort were divided into groups with high and low-risk levels by using the optimal truncation value. Subsequently, we examined the prognostic disparities between the two groups and evaluated the accuracy of the model.

### 2.5 Determination of genes with differential expression

To identify differentially expressed genes, the cutoff was established at an absolute log2 fold change (|log2FC|) greater than 1 and an adjusted P < 0.05. The stepwise-Cox analysis employed a P < 0.1 to determine the final SRGs. Paired samples from TCGA, which consisted of cancer and paraneoplastic tissues from the same individual, were employed to explore spatial differences in gene expression.

### 2.6 Analysis of immune infiltration

The infiltration of 22 immune cells was evaluated using the “CIBERSORT” algorithm. The robustness of the CIBERSORT algorithm was validated using four additional algorithms: xCell, ESTIMATE, GSVA, and MCP-counter. The immune checkpoints were derived from established research (Wang et al., 2021).

### 2.7 Analysis of SRG gene networks and enrichment

A gene network analysis using GENEMANIA (http://genemania.org/) was conducted to examine potential interactions between these genes. To investigate the mechanisms linking riskscore clusters, functional enrichment analyses, including Gene Ontology (GO), Kyoto Encyclopedia of Genes and Genomes (KEGG), Gene Set Enrichment Analysis (GSEA), and Gene Set Variation Analysis (GSVA), were performed on differentially expressed genes using R packages such as “clusterProfiler,” “enrichplot,” “limma” and “ggplot2.”

### 2.8 Clinical specimens

The study was approved by the Ethics Committee of Affiliated Hospital of Nantong University. All participants/patients participating in this study have informed consent.

#### 2.8.1 Tissue microarray construction and immunohistochemistry

Four sets of microarrays of tumor and paracancer tissues provided by the Department of Pathology, Affiliated Hospital of Nantong University, aiming to verify the accuracy of the prognosis model. The immunotissue microarrays included cancer tissues and adjacent tissues from 127 patients with lung adenocarcinoma, including 64 males and 63 females, 68 patients <65 years old, 59 patients ≥65 years old, 60 patients died, and 67 patients were still alive. Due to the loss during the chip production process, some samples fell off, so 118 LDHA chip samples, 106 CDKN3 chip samples, 112 SHC1 chip samples, and 108 BTK chip samples were finally obtained. Primary Anti-LDHA antibody (1:150; No: A18574, ABclonal, China), Anti-CDKN3 antibody (1:150; No: A2061, ABclonal, China), Anti-SHC1 antibody (1:150; No: A7725, ABclonal, China), Anti-BTK antibody (1:400; No: A19002, ABclonal, China) were used for the immunohistochemical (IHC) staining. Immunohistochemistry sections were scanned using a slide scanning system (TEKSQRAY, China). ImageJ software was used to measure the density of positive staining and the percentage of positive immunostaining cells. Two experienced pathologists were invited to verify the accuracy of the data. The final score (IHC score) was determined by multiplying the immunostaining intensity by the percentage of positive immunostaining cells.

### 2.9 Statistical analysis

All the data processing and analysis described in this article were performed utilizing R 4.0.3 software. The unpaired Student’s t-test was used for data that were normally distributed, and the Wilcoxon test was employed for data that were not normally distributed. Univariate and multivariate Cox regression analyses were employed to assess the impact of factors on LUAD prognosis. Kaplan-Meier (K-M) survival curves were constructed to evaluate survival differences, and the log-rank test was applied to determine the statistical significance of the survival time differences between the two patient cohorts. P < 0.05 was set as the threshold for statistical significance.

## 3 Results

### 3.1 Screening and analysis of sphingolipid-related genes

**FIGURE 1:**
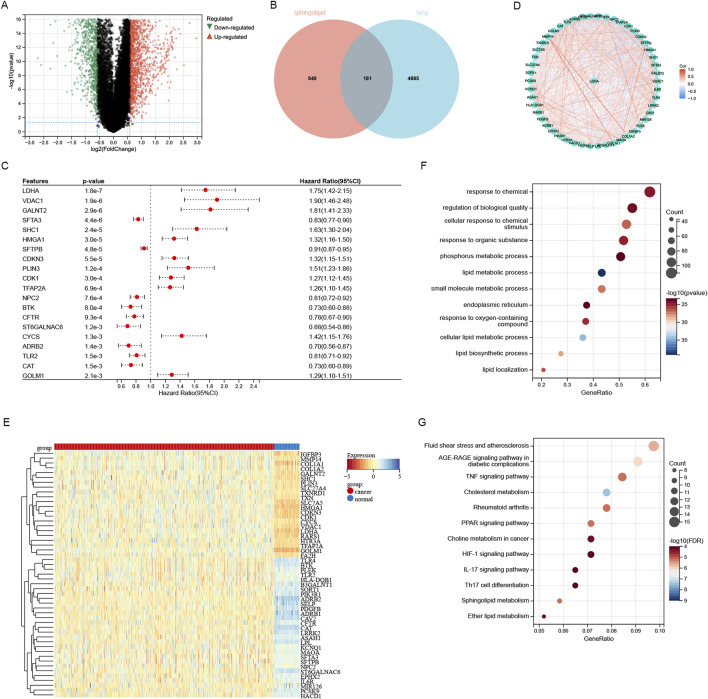
Screening and analysis of sphingolipid-related genes. **(A)** The differentially expressed genes of lung adenocarcinoma were screened through the TCGA database. **(B)** 181 sphingolipid-related genes identified by intersecting 5066 TCGA-LUAD genes and 721 GeneCards database genes. **(C)** Through univariate Cox regression analysis, 51 SRGs (P < 0.05) were identified. **(D)** Interconnection of the 51 SRGs. **(E)** Heatmap analysis about sphingolipid-related genes in LUAD versus normal tissues. **(F)** GO analysis of sphingolipid-related genes was performed. **(G)** KEGG analysis of SRGs was performed.

An analysis of the TCGA LUAD database revealed significant differential expression of 5,066 genes between tumor and normal tissues, and there were 2,850 genes that were upregulated and 2,216 genes that were downregulated in lung adenocarcinoma (Figure 1A). A total of 721 sphingolipid metabolism related genes were obtained through GENECARDS website. 181 genes were obtained by intersecting 5,066 differentially expressed genes in lung adenocarcinoma with 721 sphingolipid metabolism related genes obtained from GENECARDS. These genes are not only closely related to sphingolipid metabolism, but also show different expression levels between lung cancer and paraneoplastic tissue (Figure 1B). Univariate COX regression analysis was utilized for identifying 51 genes associated with sphingolipid metabolism (Figure 1C) (Figure 1C only listed the first 20 genes). Figure 1D illustrates the protein-protein interaction network among 51 genes related to sphingolipid metabolism. The heatmap described the expression patterns and characteristics of 51 differentially expressed genes (DEGs) in sphingolipid metabolism family, including 29 sphingolipid metabolism family genes downregulated and 22 sphingolipid metabolism family genes upregulated (Figure 1E). We investigated the primary enrichment pathways of these DEGs by analyzing the enrichment of 51 genes using KEGG and GO pathways. These results demonstrated that these genes were enriched in small molecule metabolic process, lipid metabolic process, endoplasmic reticulum and phosphorus metabolic process pathways by GO analysis. KEGG analysis primarily focused on pathways including AGE-RAGE signaling in diabetic complications, TNF signaling, Cholesterol metabolism, PPAR signaling, Choline metabolism in cancer, HIF-1 signaling, IL-17 signaling, and Th17 cell differentiation (Figures 1F, G).

### 3.2 Consensus clustering analysis of prognostic genes related to sphingolipids

**FIGURE 2:**
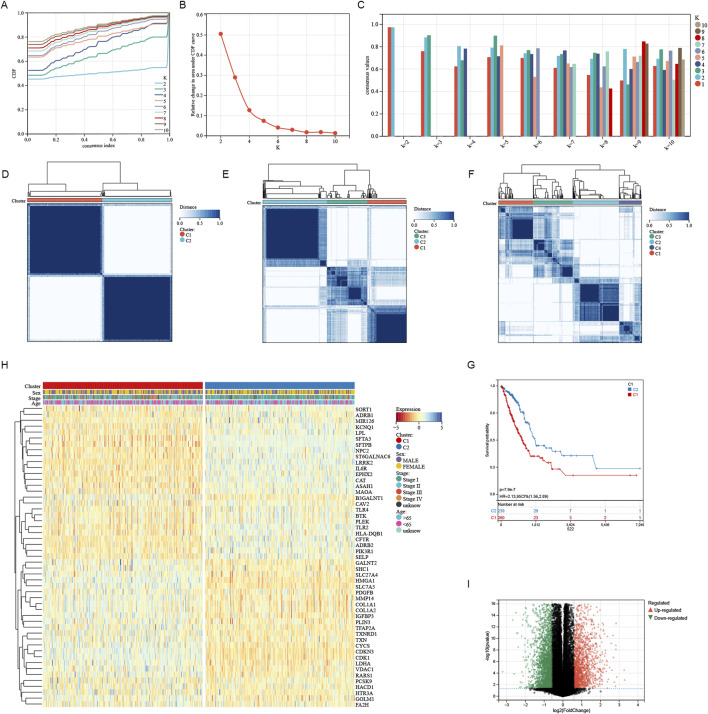
Characteristics of sphingolipid-related Cluster in TCGA-LUAD cohort. **(A)** The consensus clustering’s delta area curve demonstrated the relative shift in the area beneath the cumulative distribution function (CDF) curve for values of k from 2 to 10. **(B)** Intragroup correlations reached their maximum, while intergroup correlations were minimal when k = 2. **(C)** The consistency of sample clustering. **(D–F)** The average consistency evaluation within the cluster group shows that K = 2 has the highest consistency, with K = 3 having the second highest. **(G)** Survival probabilities for cluster1 and cluster2 were depicted by the Kaplan-Meier curve. **(H)** The heatmap illustrated contacts and differences between clinical characteristics and the expression of SRGs in two groups. **(I)** The volcano plot revealed the distinct gene expressions across the two clusters.

**FIGURE 3:**
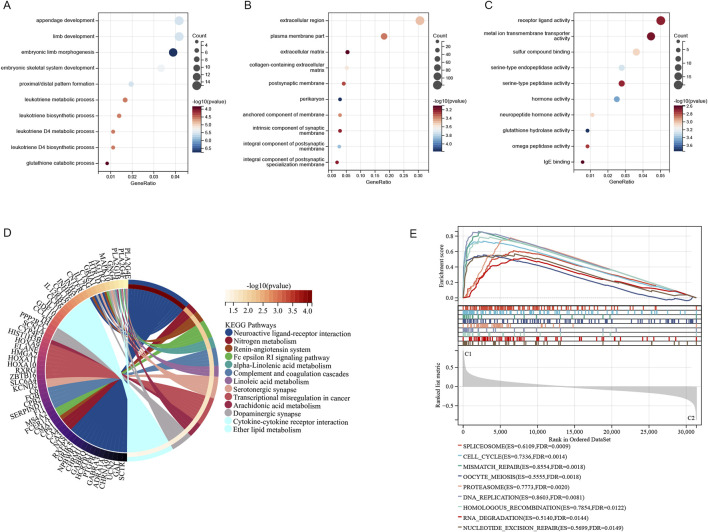
The two clusters exhibited notable enrichment in Gene Ontology (GO) categories **(A–C)**, KEGG pathways **(D)**, and multiple pathways identified through Gene Set Enrichment Analysis (GSEA) **(E)**.

A consistent cluster analysis involved in 501 cancer samples from the TCGA cohort were conducted for examining the relationship between 51 sphingolipid metabolism-related genes and LUAD subtypes. K = 2 is determined as the optimal number established on the cumulative distribution function (CDF) curve of the consensus score matrix (Figures 2A–C) and the proportion of ambiguous clustering (PAC) statistics (Figures 2D–F). Samples with high consistency scores are more likely to be repeatedly clustered together. Therefore, 442 patients were organized into two groups, with 265 in group 1 and 248 in group 2. Following this, a marked difference in OS was detected between the two groups (Figure 2G). The heatmap illustrates the correlation between different genes expression and clinical characteristics (Figure 2H). Between the two clusters, there were 851 upregulated genes and 1,414 downregulated genes, with the volcano plot depicting the logFC and FDR values of these genes (Figure 2I). According to GO enrichment analysis, we can find that these genes were connected with different molecular processes such as leukotriene metabolism, extracellular matrix, receptor-ligand interaction, and hormone activity (Figures 3A–C). The analysis of KEGG enrichment highlighted significant enrichment in pathways such as Nitrogen metabolism, Transcriptional misregulation in cancer, Neuroactive ligand-receptor interaction, alpha-Linolenic acid metabolism and Cytokine-cytokine receptor interaction (Figure 3D). GSEA enrichment analysis identified that pathways like SPLICEOSOME, CELL CYCLE, MISMATCH REPAIR, and DNA REPLICATION were enriched with differentially expressed genes in the two clusters (Figure 3E).

### 3.3 Construction and verification of the SRGs prognostic model

**FIGURE 4:**
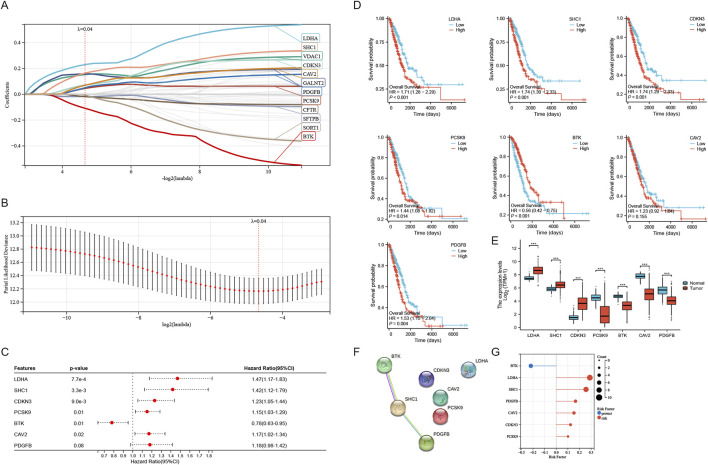
Construction of the SRGs Prognostic Model. **(A, B)** The analysis with LASSO employed the least lambda value. **(C)** The stepwise Cox algorithm was used to identify seven genes. **(D)** Prognostic curves of 7 sphingolipid metabolism related genes. **(E)** mRNA expression levels in paired TCGA samples. **(F)** Protein interaction network analysis of sphingolipid related genes. **(G)** Coefficients of seven genes associated with sphingolipid metabolism were determined through stepwise Cox regression.

For developing a prognostic model utilizing genes associated with sphingolipid metabolism. In COX univariate analysis, 51 metabolism-related genes were significantly linked to overall survival (OS) in LUAD patients. Then we performed lasso analysis on the 51 genes, twelve genes associated with sphingolipid metabolism were identified (Figures 4A, B). To construct the model, seven genes were identified through stepwise regression (Figure 4C). To establish a risk-scoring model, the subsequent algorithms are utilized: = LDHA*0.289328503921739+SHC1*0.256323270897663+CDKN3*0.123076351124556+PCSK9*0.101300068657929+CAV2*0.15265494282098+PDGFB*0.165967594013992+BTK*(-0.219105737311587). The survival curve in Figure 4D represents the prognosis for lung adenocarcinoma patients with varying expression levels of 7 SRGs. Except for the prognosis curve of CAV2, the other OS curves were significantly different (p < 0.05). Among them, LDHA, SHC1, CDKN3 were all associated with poor prognosis, while BTK was well correlated with prognosis (Figure 4D). Analysis of mRNA expression in TCGA paired samples revealed notably enhanced levels of LDHA, SHC1 and CDKN3 in cancer tissues compared to adjacent tissues, whereas PCSK9, BTK, CAV2 and PDGFB showed significantly reduced expression (Figure 4E). Figure 4F illustrates the protein-protein interaction network analysis for seven molecules. For further analysis, TCGA-LUAD patients were categorized into high risk score (n = 187) and low risk score (n = 381) subgroups determined by the optimal cutoff. The seven SRGs’ coefficients are illustrated in Figure 4G.

**FIGURE 5:**
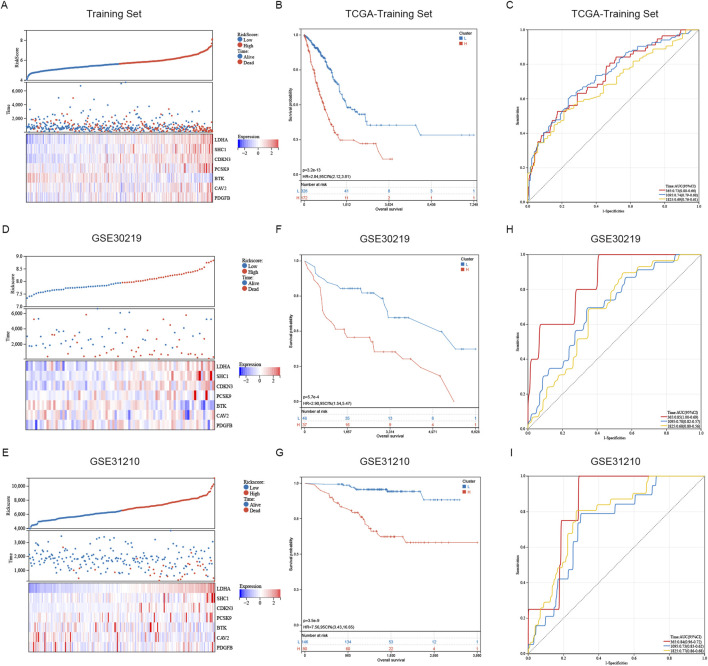
Assessment and confirmation of prognostic signature. **(A, D, E)** The risk-score, survival time, survival status and gene expression of the training set, external set GSE30219 and GSE31210. **(B, F, G)** K-m analysis verified the risk model’s prognostic significance in the TCGA training set, GSE31210 and GSE30219 cohort. **(C, H, I)** ROC curve analysis verified the risk model’s prognostic significance in the TCGA training set, GSE31210 and GSE30219 cohort.

The distribution of risk scores and the living condition of patients in the TCGA database’s high and low risk groups are depicted in Figure 5A. The survival time of patients gradually diminishes as the risk score increases, whereas the risk of death becomes higher. The Kaplan-Meier analysis indicated a notably lower survival rate for the high-risk group compared to the low-risk group (Figure 5B). The training dataset was utilized to perform ROC curve analysis, evaluating the predictive accuracy of SRGs for LUAD patient outcomes. The results indicated the AUC value of the TCGA training cohort exceeded 0.69 for 1, 3, 5 years (Figure 5C). Subsequently, GEO databases GSE30219 and GSE31210 were employed as validation sets to assess the risk model (Figures 5D–I). Similar results are observed in GSE30219 and GSE31210 as in TCGA databases (Figures 5D, E). Figures 5F, G illustrate that in GSE30219 and GSE31210, compared to the low-risk group, the high-risk group had a much lower survival rate. Additionally, ROC curve analysis revealed the AUC value of GEO test cohort exceeded 0.68 at 1, 3, 5 years (Figures 5H, I). The sphingolipid-associated prognostic model demonstrates high accuracy in predicting outcomes of lung adenocarcinoma patient.

### 3.4 Prognostic risk score distribution and stratification by clinical characteristics

**FIGURE 6:**
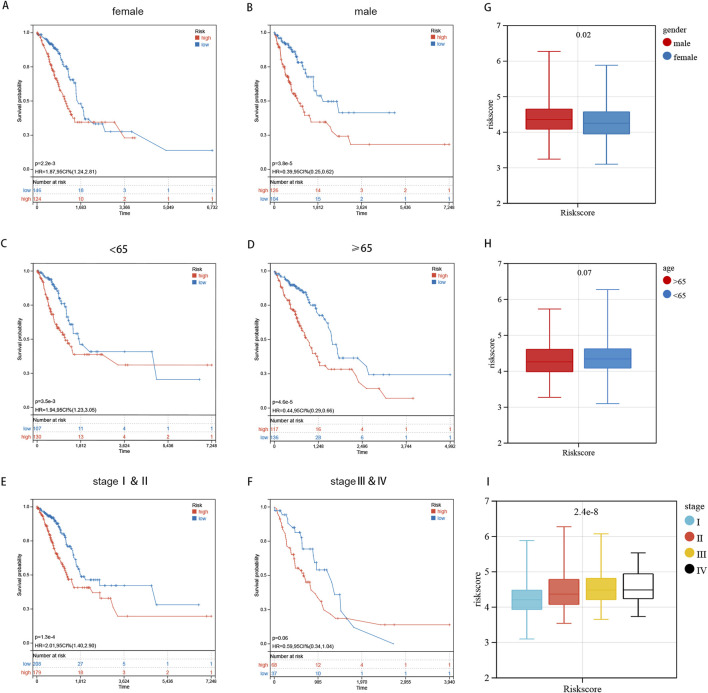
Prognostic Risk Score Distribution and Stratification by Clinical Characteristics. **(A–F)** The log-rank test and K-m curves revealed that patients at high risk had inferior overall survival outcomes compared to those at low risk, across all age and gender subgroups. **(G–I)** The distribution of risk scores is analyzed across different genders, age groups, and pathological stages.

The K-M curve revealed that high-risk samples exhibited poorer overall survival compared to the low-risk category in gender-stratified subgroups (Figures 6A, B). In both age subgroups, those over 65 and those 65 or younger, the high-risk group showed a markedly decreased overall survival rate when compared with the low-risk group (Figures 6C, D). Pathological staging and stratification revealed that in stage I and II subgroups, the prognosis of high-risk sample is worse (Figure 6E). While no significant OS difference was observed between two groups in stage IV and its subgroups, the overall OS trend remained consistent with pre-stratification results (Figure 6F). The reason why no obvious difference in OS is observed is that smaller sample size post-stratification and the decline in calculation efficiency, we speculate. The survival probability of LUAD patients in the high-risk group was consistently lower than that of the low-risk group across various clinical subgroups (Figures 6A–F). The K-m clustering subgroup shows notable distinctions in risk score distribution, confirming the consistency between K-m clustering signature results and sphingolipid metabolism (Figures 6A–F). In general, gender affects risk scores, while the female patients’ risk score is lower than that of male patients (Figure 6G). The risk score distribution did not significantly differ between the subgroups of age ≥65 and <65, indicating that age does not influence the risk score distribution (Figure 6H). The risk score increases with tumor progression and changes in pathological stage (Figure 6I). The findings indicate that, after stratifying clinical features, our risk score distribution significantly influences LUAD patient prognosis, demonstrating high specificity and sensitivity in prognostic prediction.

### 3.5 Immune infiltration analysis for sphingolipid-signature

**FIGURE 7:**
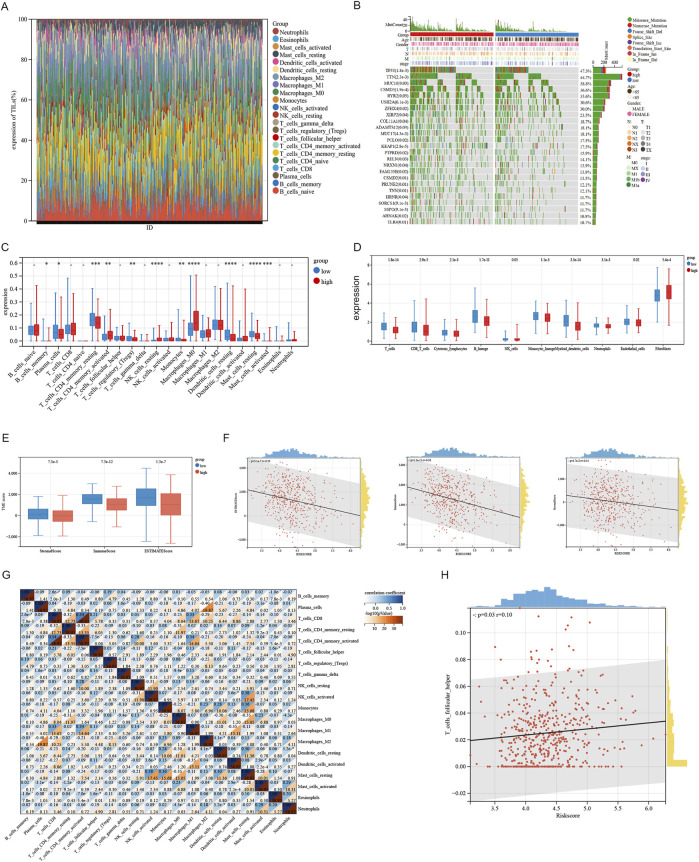
Analysis of immune infiltration associated with the signature. **(A)** The immune infiltration analysis in LUAD patients compared the percentage abundance of tumor-infiltrating immune cells between high and low risk-score groups. **(B)** Heatmap illustrating differential gene expression associated with sphingolipid-related signatures in LUAD. **(C)** The degrees of immune cell infiltration in patients with LUAD were compared across two risk groups. **(D)** Xcell algorithms identified immune cell expression differences between two risk groups. **(E, F)** Examining the differences in ESTIMATE, stromal, and immune scores between cluster 1 and cluster 2. **(G)** Correlation between immune cells. **(H)** The Riskscore is proportional to the T cells follicular helper.

We analyzed variations in the infiltration of 22 immune cell types across these SRGs subgroups utilizing the Cibersort database (Figures 7A, C). Clinical characteristics and their association with risk score subgroups are shown on the heatmap (Figure 7B). The study found that the high-risk group exhibited considerably higher levels of activated NK cells, resting macrophages M0, and activated mast cells compared to the low-risk group. On the contrary, the high-risk group had significantly lower levels of resting CD4 memory T cells, regulatory T cells (Tregs), resting dendritic cells, and resting mast cells. A significant positive association was found between the T_cells_follicular_helper and the Riskscore (Figure 7H). Demonstrating that the function of two subgroups remains unaffected by the analysis algorithm, we employed the MCP-counter (Figure 7D) and ESTIMATE (Figure 7E) algorithms to verify the accuracy and stability of the Cibersort results, which were consistent with the Cibersort database evaluations. As shown in Figure 7F, riskscore is negatively correlated with both ESTIMATEScore, ImmuneScore and StromalScore (Figure 7F). We observed a negative correlation (R = −0.4) between macrophage M2 and Plasma_cells in the lung adenocarcinoma microenvironment (Figure 5G). In summary, these findings indicate that high-risk subgroups exhibit considerably reduced immune infiltration and increased tumor purity, potentially influencing immunotherapy outcomes of LUAD patients.

### 3.6 Validation of *in vitro* experiments

**FIGURE 8:**
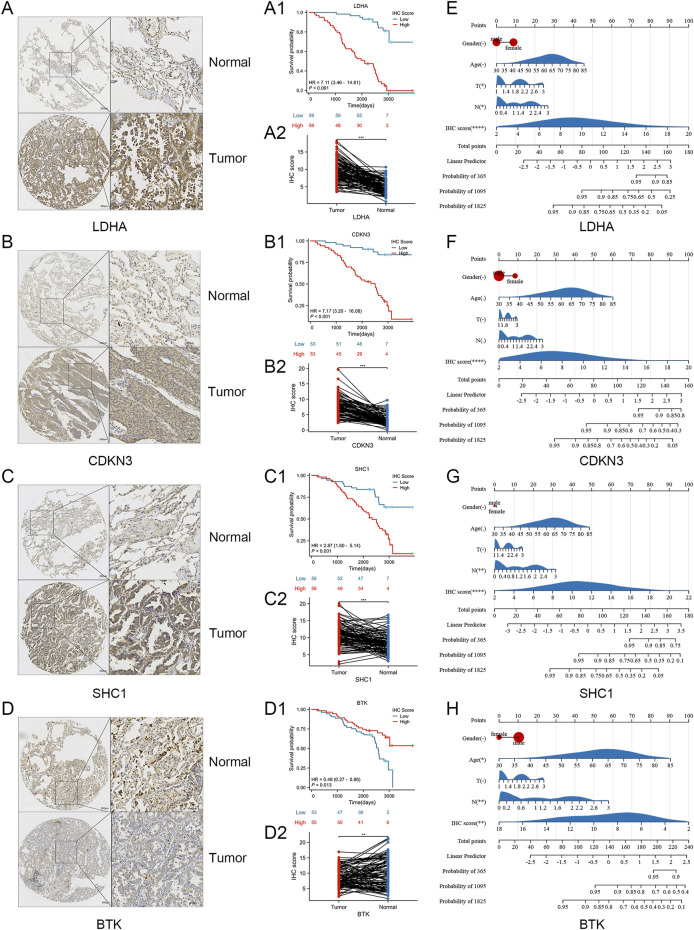
Verification of laboratory experiments. **(A–D)** Immunohistochemistry (IHC) results indicated that the protein expression of LDHA, CDKN3, SHC1 were significantly elevated in tumor tissues compared to normal tissues. And the expression of BTK protein in tumor tissues was lower compared to normal tissues. **(A1, A2)** K-m curve and nomogram of LDHA. **(B1, B2)** K-m curve and nomogram of CDKN3. **(C1, C2)** K-m curve and nomogram of SHC1. **(D1, D2)** K-m curve and nomogram of BTK. **(E–H)** The nomogram for the Nantong cohort incorporates the IHC-score alongside clinical parameters such as age, gender, and T, N stage. Significance levels: *P < 0.05, **P < 0.01, ***P < 0.001, ****P < 0.0001.

We conducted *in vitro* experiments for validating the differential expression of SRGs in cancer and adjacent tissues of LUAD. A cohort study utilized a tissue microarray from 127 lung adenocarcinoma patients at Nantong Hospital. Immunohistochemical staining was utilized for identifying the expression of LDHA, CDKN3, SHC1, and BTK in 127 lung cancer samples (Figures 8A–D). The reason why we chose these four molecules is that they showed obvious differences in the previous K-M curves (P < 0.001) (Figure 4D). By immunohistochemical staining, we can find that all four molecules are expressed in cytoplasm (Figures 8A–D). Immunohistochemical score (IHC score) of tumor tissue was different in each sample. The expression of LDHA, CDKN3, and SHC1 were significantly raised in lung adenocarcinoma compared to non-cancer cells, whereas BTK expression was higher in non-cancer than in cancer cells (Figures 8A2, B2, C2, D2). The 127 patients were categorized into two groups attributed to their IHC scores. In Nantong cohort, patients exhibiting high expression levels of LDHA, CDKN3, and SHC1 had a significantly poorer prognosis compared to those with low expression levels of these markers (Figures 8A1, B1, C1). Patients with high BTK expression have a better prognosis compared to those with low BTK expression (Figure 8D1). Despite the minor statistical difference in patients’ OS shown in Figure 8D1 (P = 0.013), the general trend aligns with the TCGA database, possibly due to the limited sample size and varying ethnic groups in the sample. Crucially, all four SRGs developed nomograms, identifying the IHC score as a key independent predictor of patient prognosis (Figures 8E–H). While age and gender do not seem to be key prognostic factors, and T and N have some predictive value. The Harrell C-index values of the nomogram models predicting overall survival rates are 0.81 for LDHA, 0.79 for CDKN3, 0.75 for SHC1, and 0.73 for BTK. In a word, our research shows that the expressions of key genes of sphingolipid metabolism, such as LDHA, CDKN3, SHC1 and BTK, are obviously different from those of non-cancerous tissues in lung adenocarcinoma, and can obviously affect the prognosis of LUAD patients.

## 4 Discussion

Cancer has consistently posed a significant challenge in modern medicine worldwide. Lung cancer is the primary cause of death from cancer in men and ranks as the second leading cause in women, following breast cancer (Bray et al., 2024). Approximately 40% of diagnosed cases are lung adenocarcinomas (LUADs) (Leiter et al., 2023). The invasion of cancer cells into adjacent tissues is primarily driven by mechanisms of immune evasion and drug resistance, which are major contributors to cancer mortality. Recent progress in molecular targeted and immunotherapies has gradually improved survival rates for LUAD patients. Despite advancements, drug resistance and recurrence are key factors in LUAD progression, primarily driven by immune evasion and resistance to tumor cell apoptosis (Zhan et al., 2019; Nechiporuk et al., 2019; Yu et al., 2023). The intricate molecular mechanisms of LUAD contribute to its unfavorable prognosis, suggesting that constructing a predictive model utilizing multiple genes could be a more efficacious approach (Laurence et al., 2021; Zhang et al., 2023b). Recently, an increasing number of prognostic markers for LUAD have been identified (Zhang et al., 2024a; Zhang et al., 2024b). For example, there may be potential links between programmed cell death caused by intestinal microorganisms and mitochondria and LUAD prognosis, diagnosis and treatment (Fang et al., 2024; Feng et al., 2024; Zhang et al., 2024c). However, the current paucity of adequate biomarkers hinders this objective, underscoring the necessity of identifying additional biomarkers for enhancing the accuracy of early predictive models in LUAD.

The cell membrane is composed of diverse lipids, among which sphingolipids are integral in preserving structural integrity of the membrane and regulating its mobility (Ogretmen, 2018). Sphingolipids belong to a broader class of lipids. Furthermore, as secondary messengers in cellular signal transduction, sphingolipids are implicated in the regulation of numerous biological processes (Dany and Ogretmen, 2015; Pettus et al., 2005). Recent studies found a strong link between sphingolipid metabolism and tumor development and progression, influencing processes such as cell death, senescence, cell cycle arrest, cell proliferation, and anti-apoptosis (Obeid et al., 1993; Lee et al., 1998; Hannun et al., 2015; Morad and Cabot, 2013).

We initially screened 51 genes associated with sphingolipid metabolism in lung adenocarcinoma tissues, comparing them to normal tissues. Among these, 29 genes exhibited downregulation, while 22 genes were upregulated. Subsequently, we employed the K-m algorithm to categorize TCGA samples into two distinct clusters. Differentially expressed genes in the two LUAD clusters are mainly enriched in processes related to phosphorus and lipid metabolism, small molecule metabolism, and the endoplasmic reticulum. They are also associated with pathways such as TNF signaling, cholesterol metabolism, PPAR signaling, choline metabolism in cancer, IL-17 signaling, HIF-1 signaling, and Th17 cell differentiation. The overall survival (OS) in cluster 1 was worse than in cluster 2. These findings indicate that the prognostic differences among clusters related to sphingolipid metabolism are associated with variations in immune response. Seven SRGs were identified using univariate regression, LASSO, and stepwise regression analyses. And these SRGs were used to create new prognostic risk profiles, enabling the classification of LUAD patients into high-risk and low-risk groups. The high-risk group had a significantly poorer prognosis than the low-risk group. We evaluated the model’s accuracy by performing comprehensive ROC curve analyses on the training and test cohorts. The AUC values surpassed 0.68 at 1, 3, and 5 years, peaking at 0.74 at 3 years. The differential expression of mRNA related to sphingolipid metabolism was confirmed using paired samples from TCGA. Subsequently, the protein expression of sphingolipid metabolism was further confirmed through immunohistochemical experiments. The prognostic signals were integrated with 7 SRGs, including LDHA, SHC1, CDKN3, PCSK9, CAV2, PDGFB and BTK. Among them, LDHA, SHC1 and CDKN3 were associated with poor prognosis, while BTK was well correlated with good prognosis. Finally, we selected four molecules LDHA, CDKN3, SHC1 and BTK, which are most significantly impacting the prognosis of patients, for *in vitro* experiments. In Nantong cohort, our analysis revealed that LDHA, SHC1, and CDKN3 are associated with poor prognosis, whereas BTK is linked to favorable prognosis, aligning with findings from the TCGA database. We created a nomogram and found the immunohistochemical (IHC) score to be a key independent predictor of overall survival.

Prior studies have descripted a negative correlation between tumor purity and immune response, indicating that tumor purity could be an indicator of immune response levels in the tumor microenvironment (Liu et al., 2019). Similar to these findings, our study demonstrated that immune infiltration was lower in the high-risk group than in the low-risk group. Significant differences were observed in the abundance of immune cell types, such as activated CD4 memory T cells, M0 macrophages, resting mast cells, resting natural killer corpuscles, resting dendritic cells, activated mast cells and resting CD4 memory T cells between the two groups. The critical environment for cancer development is composed of the tumor microenvironment (TME) (Mantovani et al., 2008; Engblom et al., 2016) which leading to tumor immune escape (Li et al., 2024; Wang et al., 2022). And the relationship between sphingolipid metabolism and signal transduction has received increasing attention in the tumor microenvironment. Sphingolipids play a crucial role in regulating inflammation and extracellular matrix dynamics in the tumor microenvironment. For example, S1P can activate various signaling pathways in immune cells, endothelial cells, and fibroblasts, affecting the secretion of cytokines, chemokines, and growth factors that regulate inflammation (Mohammed et al., 2023; Weigel et al., 2023). By regulating the tumor immune microenvironment, S1P affects tumor progression and the effectiveness of immunotherapy. For example, in breast cancer, the S1P gradient between peripheral blood and bone marrow mediates the redistribution of Treg cells (Rathinasamy et al., 2017). Ceramide is an essential component of T cell immune development. In mouse models, inhibition of ceramide synthesis has been shown to promote the differentiation of immunosuppressive Treg cells and impair the function of cytotoxic T cells (Hose et al., 2022). Ceramide can activate the T cell receptor (TCR) and co-stimulatory molecule CD28 on the surface of T cells, thereby enhancing the activation of CD8^+^ T cells (Vaena et al., 2021). Cumin et al.’s study showed that sphingolipids contribute to the remodeling of extracellular matrix (ECM) vascular structure, thereby affecting the proliferation, invasion and metastasis of cancer cells (Cumin et al., 2022). Current research suggests that innate lymphocytes (ILC) in the TME could hinder the initiation and advancement of digestive system tumors and impact the effectiveness of immunotherapy (Shen et al., 2024). Inflammatory cells and mediators represent essential elements of the TME, with tumor-associated macrophages (TAMs) exemplifying the connection between inflammation and oncogenesis (Mantovani et al., 2017). The primary reason for mortality from tumors, such as lung cancer, is metastasis (Ruixin et al., 2024). In most tumors, macrophages aid cancer progression and metastasis by supporting cancer cell survival and growth, promoting angiogenesis, and inhibiting innate and adaptive immune responses (Coussens et al., 2013; Cassetta and Pollard, 2018; Locati et al., 2020; Murray et al., 2014). Our analysis demonstrated significantly higher expression levels of M0 macrophages in the high-risk group compared to the low-risk group, consistent with prior observations in renal clear cell carcinoma (Farha et al., 2023).

Our study integrates data from two large public databases, TCGA and GEO, and constructs a prognostic model based on 7 sphingolipid-related genes, breaking through the limitations of single database analysis and improve the reliability and generalization capabilities of the model. In addition, we verified the functions of key genes through *in vitro* experiments, further supporting the biological rationality of the model. Immune infiltration analysis further illustrates that the model is Value in immunotherapy, the expression level of M0 macrophages in the high-risk group is significantly increased, so we may be more effective in using M0 macrophage-related immunotherapy drugs for the treatment of high-risk patients. Our model can provide clinicians with Prognostic information on patient survival probability and disease progression helps develop personalized treatment options. Risk stratification of patients to enable closer monitoring and more active treatment of high-risk patients while avoiding overtreatment of low-risk patients, reduces the medical costs of patients. As well as provide advice on patient education, psychological support, and clinical research. However, there are still some areas that need improvement. In the future, our research needs to further integrate multi-omics data, use deep learning algorithms and artificial intelligence technology, and build a more comprehensive prognostic model to more accurately reflect the biological characteristics of the tumor and disease progression. And verify the accuracy and reliability of the prognostic model in more independent clinical cohorts, and further optimize the model based on feedback.

This study aims to identify a biomarker linked to sphingolipid metabolism for predicting survival outcomes in lung adenocarcinoma (LUAD) patients and to explore the effects of tumor immune infiltration and immunotherapy on prognosis. It is anticipated that our findings will offer novel insights into the precise diagnosis and treatment strategies for LUAD patients.

## Data Availability

The datasets presented in this study can be found in online repositories. The names of the repository/repositories and accession number(s) can be found below: https://www.ncbi.nlm.nih.gov/, The Cancer Genome Atlas (TCGA) database https://www.ncbi.nlm.nih.gov/geo/, GSE31210 https://www.ncbi.nlm.nih.gov/geo/, GSE30219.
